# Preparation and Photocatalytic Properties of Al_2_O_3_–SiO_2_–TiO_2_ Porous Composite Semiconductor Ceramics

**DOI:** 10.3390/molecules29184391

**Published:** 2024-09-15

**Authors:** Kaihui Hua, Zhijing Wu, Weijie Chen, Xiuan Xi, Xiaobing Chen, Shuyan Yang, Pinhai Gao, Yu Zheng

**Affiliations:** 1 School of Environment and Civil Engineering, Dongguan University of Technology, Dongguan 523808, China; wuzhijing-2001@foxmail.com (Z.W.); c13266257357@163.com (W.C.); chen_xb4869@163.com (X.C.); gaoph@dgut.edu.cn (P.G.); zhengy@dgut.edu.cn (Y.Z.); 2 Guangdong Provincial Key Laboratory of Intelligent Disaster Prevention and Emergency Technologies for Urban Lifeline Engineering, Dongguan 523808, China; 3 School of Physical Sciences, Great Bay University, Dongguan 523000, China; 4 School of Materials Science and Engineering, Dongguan University of Technology, Dongguan 523808, China; yangsy@dgut.edu.cn

**Keywords:** porous ceramics, photodegradation, methylene blue, composite semiconductor

## Abstract

Titanium dioxide (TiO_2_) is widely employed in the catalytic degradation of wastewater, owing to its robust stability, superior photocatalytic efficiency, and cost-effectiveness. Nonetheless, isolating the fine particulate photocatalysts from the solution post-reaction poses a significant challenge in practical photocatalytic processes. Furthermore, these particles have a tendency to agglomerate into larger clusters, which diminishes their stability. To address this issue, the present study has developed Al_2_O_3_–SiO_2_–TiO_2_ composite semiconductor porous ceramics and has systematically explored the influence of Al_2_O_3_ and SiO_2_ on the structure and properties of TiO_2_ porous ceramics. The findings reveal that the incorporation of Al_2_O_3_ augments the open porosity of the ceramics and inhibits the aggregation of TiO_2_, thereby increasing the catalytic site and improving the light absorption capacity. On the other hand, the addition of SiO_2_ enhances the bending strength of the ceramics and inhibits the conversion of anatase to rutile, thereby further enhancing its photocatalytic activity. Consequently, at an optimal composition of 55 wt.% Al_2_O_3_, 40 wt.% TiO_2_, and 5 wt.% SiO_2_, the resulting porous ceramics exhibit a methylene blue removal rate of 91.50%, and even after undergoing five cycles of testing, their catalytic efficiency remains approximately 83.82%. These outcomes underscore the exceptional photocatalytic degradation efficiency, recyclability, and reusability of the Al_2_O_3_–SiO_2_–TiO_2_ porous ceramics, suggesting their substantial potential for application in the treatment of dye wastewater, especially for the removal of methylene blue.

## 1. Introduction

TiO_2_ is extensively used in environmental applications, such as hydrogen production [1], catalytic degradation of wastewater [2], and air purification [3]. The popularity of TiO_2_ is due to its strong stability, nontoxicity, high photocatalytic efficiency, low cost, lack of secondary pollution, and robust synthesis [4,5]. In the field of photocatalysis, most studies have focused on TiO_2_ in its powder form, mainly because powdered TiO_2_ offers a larger surface area, which is essential for enhancing photocatalytic reactions. The fine particles create more active sites for the photocatalytic process, which increases the overall efficiency of TiO_2_. Consequently, many studies have focused on optimizing the size and dispersion of TiO_2_ particles to maximize their effectiveness, especially in environmental remediation and water treatment processes [6,7,8]. However, in actual photocatalysis processes, separating the fine powdery photocatalysts from the solution after the reaction is challenging [9,10]. Additionally, the particles tend to aggregate into larger particles, leading to reduced stability [11,12]. Consequently, researchers are investigating suitable carriers to support the photocatalysts. For example, Wang et al. [10] employed an alkalization/dealkalization and wet spinning process to synthesize TiO_2_@Ti_3_C_2_T_x_ fibers, which demonstrated effective dye degradation. Their approach expanded the d-spacing of Ti_3_C_2_T_x_ layers within the microfibers, which consequently promoted the growth of TiO_2_ nanoparticles. The resulting TiO_2_@Ti_3_C_2_T_x_ fibers demonstrated rapid RhB dye degradation and excellent recyclability in water. He et al. [13] developed porous ZrO_2_/Al_2_O_3_ ceramic bead carriers loaded with BiOI photocatalyst via gel-dripping and solvothermal methods. The supported BiOI catalyst prepared at 160 °C for 6 h achieved a high degradation rate of 98.5%. The supported BiOI catalyst showed superior recyclability than the standalone BiOI powder. This approach effectively addresses the recovery challenges associated with powder catalysts.

Porous ceramics are widely used in various applications, including catalyst support, sound absorption, and heat insulation. They are favored for their high porosity, large specific surface area, excellent chemical stability, and strong thermal shock resistance [14,15,16]. For example, Liao [17] prepared porous SiC ceramic supports loaded with catalysts. The catalytic performance remained stable, with no significant deactivation observed after 30 h of reaction. This approach effectively addressed the recovery issues associated with powder catalysts through carrier loading. Thus, using porous ceramics as catalyst carriers presents a promising solution to the stability and recovery challenges of TiO_2_ in photocatalysis. Traditionally, fabricating TiO_2_-coated porous ceramics involves a method that requires multiple soakings in a TiO_2_ solution followed by high-energy, carbon-intensive sintering [15]. This process not only consumes a significant amount of energy but also raises concerns about water shock performance, which limits the development and application of the technology. Therefore, the preparation of photocatalysts in ceramic form is being reconsidered.

Although ceramic TiO_2_ can improve the recovery efficiency and stability of TiO_2_, its overall photocatalytic activity is often more reduced than that of dispersed TiO_2_. This reduction is owing to the lower surface-to-volume ratio and partial loss of active sites on the photocatalyst surface [18,19]. To address these issues, TiO_2_ can be combined with insulators that have a large specific surface area and a good pore structure. These insulators are chemically inert, have high surface areas, and possess unique surface properties that enable uniform dispersion of various semiconductor bases and enhance the exposure of active sites during photocatalysis. For example, Mandal et al. [20] prepared silica nanosphere composites loaded with Fe_2_O_3_ nanoparticles using the sol-gel method. The results showed that the uniform distribution of hematite particles on the silica surface enhanced catalytic activity. The optimization of the loading to 20% hematite nanoparticles led to minimized agglomeration, maximized catalytic sites, and overall optimal performance. Additionally, insulators can optimize the optical path and improve light scattering efficiency, which enhances photon capture in insulator-based photocatalysts and promotes the generation of light-induced carriers [21]. In semiconductor-insulator composites, TiO_2_ is often combined with Al_2_O_3_, SiO_2_, and other insulators [22,23]. Al_2_O_3_ is commonly used as a primary component for catalyst carriers owing to its ability to provide a good pore structure and specific surface area while inhibiting catalyst agglomeration at high temperatures [24]. For example, Magnone E [25] observed that the photocatalytic degradation activity of a TiO_2_/Al_2_O_3_ mixture prepared using the sol-gel method was higher than that of pure TiO_2_. This led to the development of TiO_2_-supported Al_2_O_3_ ceramic hollow fibers with high photocatalytic stability. SiO_2_ not only provides a good pore structure and specific surface area but also increases the acid content and hydroxyl number on the surface of ceramics, thus improving their mechanical strength [26,27]. Additionally, owing to its light scattering effect, SiO_2_ enhances the exposure of the photocatalyst to incident light and improves light energy utilization. For example, Wang et al. [28] prepared a TiO_2_/SiO_2_ composite material that significantly improved the photocatalytic degradation activity of TiO_2_ on methyl orange compared with bare titanium dioxide and P25. In this composite, SiO_2_ enhanced the exposure of titanium dioxide to ultraviolet radiation and generated numerous photoexcited carriers that improved the photocatalytic performance of the TiO_2_/SiO_2_ catalyst.

In this study, porous semiconductor ceramics composed of Al_2_O_3_-SiO_2_-TiO_2_ were fabricated using the dry pressing technique. Anatase-phase TiO_2_ was selected as the primary catalyst due to its superior photocatalytic performance, which is attributed to its larger specific surface area and more efficient electron–hole pair separation. Al_2_O_3_ and SiO_2_ were incorporated as additive materials to enhance the ceramic matrix, and corn starch served as a pore-forming agent. The influence of Al_2_O_3_ and SiO_2_ on the structure and properties of TiO_2_ porous ceramics was systematically investigated. The results found that at an optimal composition of 55 wt.% Al_2_O_3_, 40 wt.% TiO_2_, and 5 wt.% SiO_2_, the resulting porous ceramics exhibit exceptional photocatalytic degradation efficiency, recyclability and reusability, suggesting their substantial potential for application in the treatment of dye wastewater.

## 2. Results and Discussion

### 2.1. Effect of Al_2_O_3_ Content on Ceramic Performance and Structure

**Figure 1:**
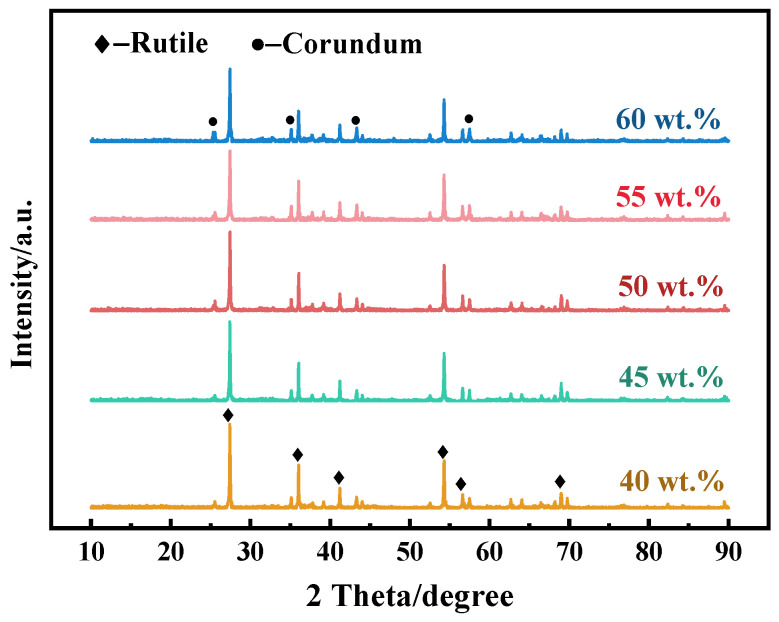
XRD patterns of porous ceramics with Al_2_O_3_ content of 40 wt.%, 45 wt.%, 50 wt.%, 55 wt.%, and 60 wt.%.

The structural analysis of the porous ceramics was first performed by XRD analysis. The crystalline phase of samples with varying amounts of Al_2_O_3_ was determined by X-ray powder diffraction analysis (Figure 1). The patterns revealed that the sample powder mainly consisted of the rutile phase (TiO_2_, PDF#21-1276) and the corundum phase (Al_2_O_3_, PDF#46-1212), indicating the complete conversion of anatase to rutile in the samples. This phase transition phenomenon is consistent with the findings of Soylu [29] and Yang [30], where anatase TiO_2_ also fully converted to the rutile phase at temperatures exceeding 1000 °C in the presence of Al_2_O_3_. Notably, as the Al_2_O_3_ content gradually increased, the intensity of the corundum phase diffraction peak increased, whereas the intensity of the rutile phase diffraction peak decreased. This is consistent with the fact that the phase transition temperature from anatase to rutile on the TiO_2_-Al_2_O_3_ surface is significantly higher than that of pure TiO_2_ [24,31]. Therefore, at higher Al_2_O_3_ concentrations, the interaction between Al_2_O_3_ and TiO_2_ was enhanced, especially at the interface where the two phases come into contact, which decreases the surface mobility of the TiO_2_ and hinders the nucleation and growth of rutile phases [32].

**Figure 2:**
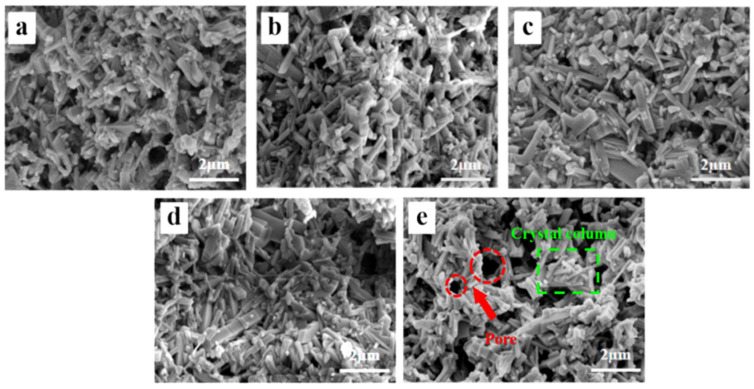
Fracture surface SEM images of porous ceramics with different Al_2_O_3_ contents: (**a**) 40 wt.%, (**b**) 45 wt.%, (**c**) 50 wt.%, (**d**) 55 wt.%, and (**e**) 60 wt.%.

Figure 2 shows the surface micromorphologies of samples with varying Al_2_O_3_ contents. The SEM images show that as the Al_2_O_3_ content increased, crystal columns began to form on the sample surface. At lower Al_2_O_3_ content levels, the surface structure exhibited agglomerative growth, which resulted in a reduction in sample pore density. As the Al_2_O_3_ content gradually increased, the crystal columns became interwoven, which led to an increase in the pore space of the sample. This phenomenon can be attributed to the regulatory effect of Al_2_O_3_ on TiO_2_ crystal growth. Specifically, Al_2_O_3_ restricted the excessive growth of TiO_2_ grains [33] while promoting the directional growth of TiO_2_ crystals at high temperatures, thereby preventing the formation of blocky structures [34]. Additionally, the presence of Al_2_O_3_ enhanced the dispersion and surface mobility of TiO_2_ by inhibiting TiO_2_ aggregation [35], resulting in a more open and porous structure on the sample surface.

**Figure 3:**
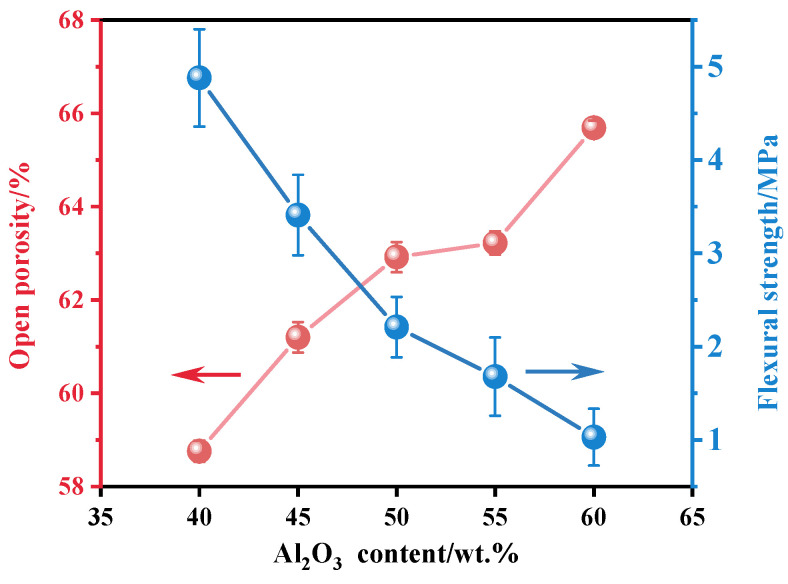
Open porosity and flexural strength of porous ceramics with Al_2_O_3_ contents of 40 wt.%, 45 wt.%, 50 wt.%, 55 wt.%, and 60 wt.%.

The open porosity and flexural strength of the ceramics are illustrated in Figure 3. As the Al_2_O_3_ content increased, the porosity of the ceramics gradually rose, and the flexural strength decreased. This trend is consistent with results from previous studies [36]. Specifically, at an Al_2_O_3_ content of 60 wt.%, the ceramic exhibited the highest porosity (65.69%) but had a flexural strength of 1.03 MPa. This is explained by Al_2_O_3_ inhibiting the agglomeration of TiO_2_ particles, leading to the gradual expansion of voidage in the sample, thereby reducing the density of the ceramic, increasing the porosity, and weakening the bending strength. Therefore, at high Al_2_O_3_ content, the mechanical properties of ceramics were significantly reduced [37].

### 2.2. Effect of SiO_2_ Content on Ceramic Performance and Structure

To address the low mechanical strength of the ceramics, SiO_2_ was introduced to fill defects with liquid phase at high temperatures, which enhanced the mechanical properties of the ceramics. Additionally, SiO_2_ increased the acid content on the ceramic surface and refined TiO_2_ grains, which enhanced the photocatalytic activity of TiO_2_.

**Figure 4:**
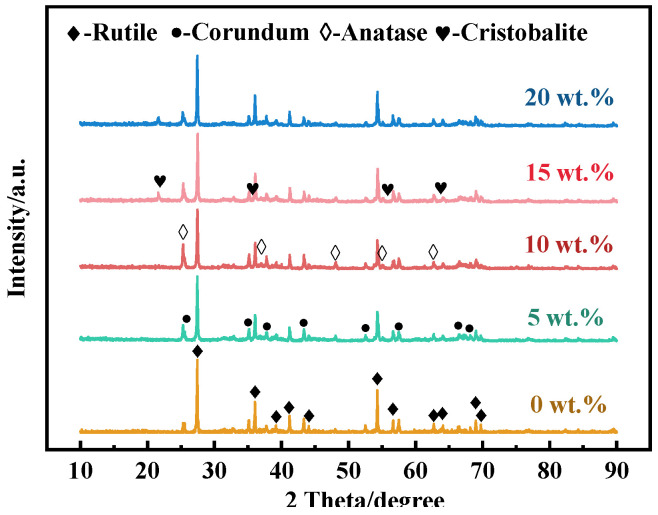
XRD patterns of porous ceramics with SiO_2_ contents of 0 wt.%, 5 wt.%, 10 wt.%,15 wt.%, and 20 wt.%.

Figure 4 displays the XRD patterns of samples with varying amounts of SiO_2_ added. In the samples without SiO_2_, only rutile and corundum phases were present. However, when SiO_2_ was added to TiO_2_, the conversion of TiO_2_ from anatase to rutile was inhibited. At 5 wt.% SiO_2_, the diffraction peak of the anatase phase (TiO_2_, PDF#21-1272) appeared, though its intensity was low. As the SiO_2_ content increased, the rutile peak intensity decreased while the anatase peak broadened, indicating that silica can suppress the anatase-to-rutile transformation and reduce the crystallite size of TiO_2_ well. The decrease in grain size can be attributed to the segregation of SiO_2_ at the grain boundaries or the presence of Ti-O-Si bonds [38]. When the content of SiO_2_ increased further, the intensity of the anatase peak weakened, and the rutile peak intensity increased. At 15 wt.% SiO_2_, the diffraction peak of the crystalline cristobalite phase (SiO_2_, PDF#27-0605) appeared, the diffraction peak intensity of the rutile phase increased, and the diffraction peak intensity of the anatase phase decreased. This transformation is consistent with the experimental results of Okada [39] and Hirano [40]. Therefore, at low SiO_2_ concentrations, SiO_2_ existed in an amorphous form within the sample. Once at higher concentrations, the solubility limit of SiO_2_ in TiO_2_ was exceeded, resulting in the formation of crystalline cristobalite.

Both anatase and rutile are tetragonal, and the A-R phase transition involves the re-stacking of TiO_6_ octahedra in the unit cell. Thermodynamically, when the grain size of anatase exceeds the critical size, the trend of the A-R phase transition is significantly enhanced [41]. In this experiment, the doping of SiO_2_ effectively inhibited this phase transition. This inhibition can be attributed to several factors: First, the incorporation of SiO_2_ led to TiO_2_ grain refinement, increased the grain boundary area, and then inhibited the growth of the rutile phase [42]. It was difficult for the smaller grain size to reach the critical size of the A-R phase transition, preventing the transition of anatase to rutile [43]. Secondly, SiO_2_ segregated at grain boundaries, limiting the migration of grain boundaries and slowing down the dynamic process of phase transition [44]. As the SiO_2_ content increased further and exceeded the solubility limit in TiO_2_, cristobalite formed. Cristobalite formation promoted heterogeneous nucleation and reduced the overall nucleation rate, which led to the A-R phase transition and a decrease in the intensity of the anatase phase diffraction peaks.

**Figure 5:**
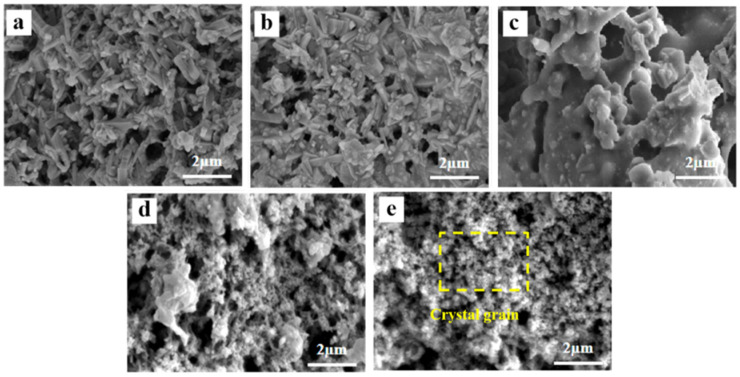
Fracture surface SEM images of porous ceramics with different SiO_2_ contents: (**a**) 0 wt.%, (**b**) 5 wt.%, (**c**) 10 wt.%, (**d**) 15 wt.%, (**e**) 20 wt.%.

The fracture surface SEM images of samples with varying SiO_2_ contents are shown in Figure 5. The surface morphology changed with increasing SiO_2_ content and affected both pore size and crystal structure. First, with increasing SiO_2_ content, the pores decreased and then increased, and the crystal columns transformed into grains. At 0 wt.% SiO_2_, the surface was predominantly composed of crystal columns. At 5 wt.% SiO_2_, the surface structure displayed agglomerative growth, with a reduction in pore size as the liquid phase formed by SiO_2_ at high temperatures filled the pores [45]. At 10 wt.% SiO_2_, further aggregation occurred, leading to reduced grain boundaries and enlarged pores. At 15 wt.% SiO_2_, the grains on the surface refined rapidly, and this refinement continued with increasing SiO_2_ content. The surface microstructure confirmed that SiO_2_ doping can refine the grain size of TiO_2_ and has a significant effect on the inhibition of the A-R phase transformation.

**Figure 6:**
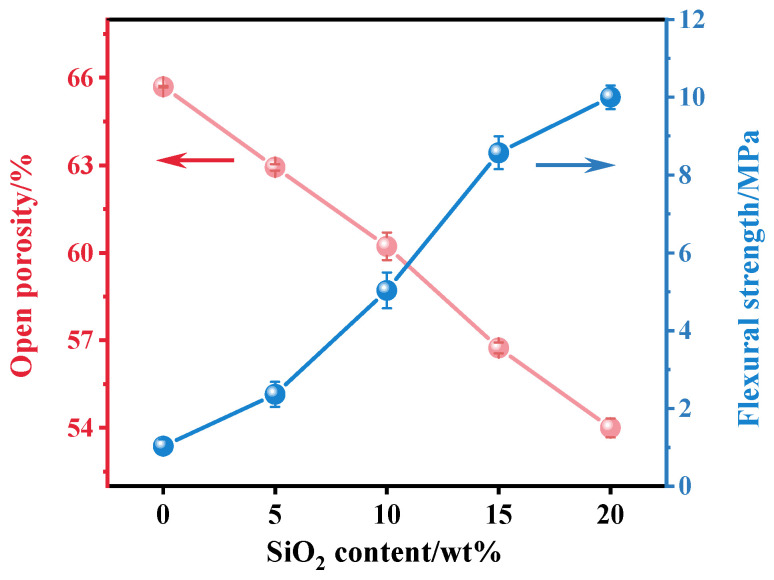
Porosity and flexural strength of porous ceramics with SiO_2_ contents of 0 wt.%, 5 wt.%, 10 wt.%, 15 wt.%, and 20 wt.%.

Figure 6 illustrates that the porosity of the sample decreased as the SiO_2_ content increased. At 0 wt.% SiO_2_, the sample exhibited the highest porosity of 65.69%, whereas at 20 wt.% SiO_2_, the porosity dropped to its lowest at 54%. This reduction in porosity can be attributed to the role of SiO_2_ during the sintering process, where SiO_2_ formed a liquid phase at high temperatures, filling the internal pores of the material and creating a denser structure. These findings are consistent with XRD and SEM analyses, which indicated that the presence of SiO_2_ reduces internal defects within the material. Furthermore, the flexural strength of the samples shows an inverse relationship with porosity, increasing with higher SiO_2_ content. At 20 wt.% SiO_2_, the flexural strength reached 10 MPa, which was substantially higher than the 1.03 MPa observed at 0 wt.% SiO_2_. This enhancement in strength can be explained by the reduction in pore-related defects due to SiO_2_ filling, leading to a more uniform and robust material structure. Additionally, this result is consistent with the findings of Zhang [46], where the addition of SiO_2_ was shown to significantly improve mechanical strength by reducing internal defects.

### 2.3. Photocatalytic Properties of Al_2_O_3_–SiO_2_–TiO_2_ Porous Ceramics

**Figure 7:**
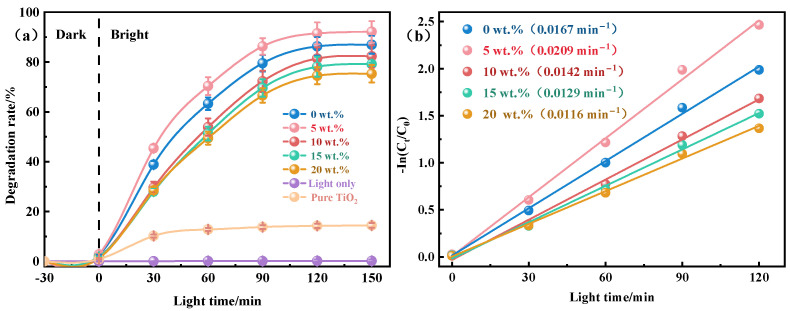
(**a**) Degradation rate and (**b**) kinetic linear simulation curve of methylene blue in simulated wastewater treated with porous ceramics of varying SiO_2_ content and pure TiO_2_ under visible light irradiation.

The photocatalytic degradation activity of the synthesized materials was studied. The preliminary control experiments were carried out to investigate the individual effects of the catalyst and visible light irradiation separately. It was found that there was no change in methylene blue concentration when the catalyst was used without irradiation, indicating that it was not activated without light. Similarly, irradiation with visible light alone was also insufficient to bring about the degradation of methylene blue. However, an appreciable decrease in methylene blue concentration was observed when the solution was exposed to visible light irradiation in the presence of the catalyst. The degradation of methylene blue was recorded in terms of percent degradation and plotted against time, as shown in Figure 7a. It was observed that the percent degradation increased by increasing the duration of light irradiation and then became nearly constant after a specific time due to the completion of the degradation process. The initial abrupt increase in photodegradation efficiency was attributed to the fact that at the start of the photocatalytic reaction, numerous catalytic sites were available for the catalytic process. Over time, the catalytic sites became occupied, and photodegradation slowed down and eventually reached a constant rate. From the graphical illustration, the degradation rate of methylene blue increased initially with SiO_2_ content but then decreased. At 5 wt.% SiO_2_, the ceramic catalyst achieved the highest degradation rate of 91.50% after 120 min of visible light exposure. It was revealed that composite insulators can optimize the surface structure and enhance light absorption, thus improving the photocatalytic activity [47]. In the present work, SiO_2_ doping has a significant impact on the surface morphology and pore structure of the materials. At a suitable doping amount (such as 5 wt.%), SiO_2_ helped to form an ideal surface pore structure, which was not only conducive to the adsorption of dye molecules, but also promoted the interaction between reactants and photogenerated carriers, thus improving the photocatalytic activity [48]. Additionally, XRD analysis showed that the incorporation of SiO_2_ promoted the formation of biphase TiO_2_. TiO_2_ has distinct crystal structures and typically exists in three crystal forms: anatase, rutile and plate titanite [49]. Due to the delay in recombining holes and electrons, anatase TiO_2_ has the highest photocatalytic activity [50]. Studies have shown that the combination of anatase and rutile phase in an appropriate proportion has better photocatalytic activity than the combination of anatase or rutile alone [51,52]. Therefore, the addition of SiO_2_ enhanced the photocatalytic activity of the sample. However, excessive SiO_2_ reduced the photocatalytic activity of the samples. There are two primary reasons for this decline. First, excessive SiO_2_ incorporation significantly reduced the porosity of the sample and water absorption, thereby reducing the adsorption performance and specific surface area of the sample. Second, when SiO_2_ exceeded the solubility limit in TiO_2_, its liquid phase promoted heterogeneous nucleation and reduced nucleation energy, which facilitated the A-R phase transition. In addition, the photocatalytic activity of pure TiO_2_ was significantly lower, and its degradation rate was lower than 15%, which further confirmed that the introduction of Al_2_O_3_-SiO_2_ plays a crucial role in improving the photocatalytic performance of TiO_2_.

Figure 7b illustrates the photodegradation kinetics of methylene blue dyes over ceramic photocatalysts with varying SiO_2_ contents. It can be seen that the plot of −ln(C_t_/C_0_) ad irradiation time (t), which provided a straight line with an R_2_ value approaching 1, indicated the excellent fit of kinetic data over the pseudo-first-order kinetic model, suggesting that the rate of photocatalytic reaction is proportional to the fraction of the catalyst’s surface interacting with the methylene blue [53]. The reaction rate for methylene blue degradation first increased with higher SiO_2_ content but then decreased. At 0 wt.% SiO_2_, the reaction rate was 0.0167 min^−1^, whereas at 5 wt.% SiO_2_, the rate increased to 0.0209 min^−1^. This phenomenon can be attributed to the effect of SiO_2_ doping on the optimization of the catalyst surface structure and the inhibition of crystal phase transformation. The more active sites exposed, the stronger the photocatalytic activity and the faster the reaction rate. Therefore, the materials doped with 5 wt.% SiO_2_ exhibited a higher reaction rate. Table 1 lists various porous ceramic catalysts used for the photocatalytic degradation of methylene blue and compares the photocatalytic efficiency of the prepared materials with previously reported materials. According to the comparison, Al_2_O_3_–SiO_2_–TiO_2_ porous ceramics are highly effective photocatalysts for degrading organic dyes.

**Figure 8:**
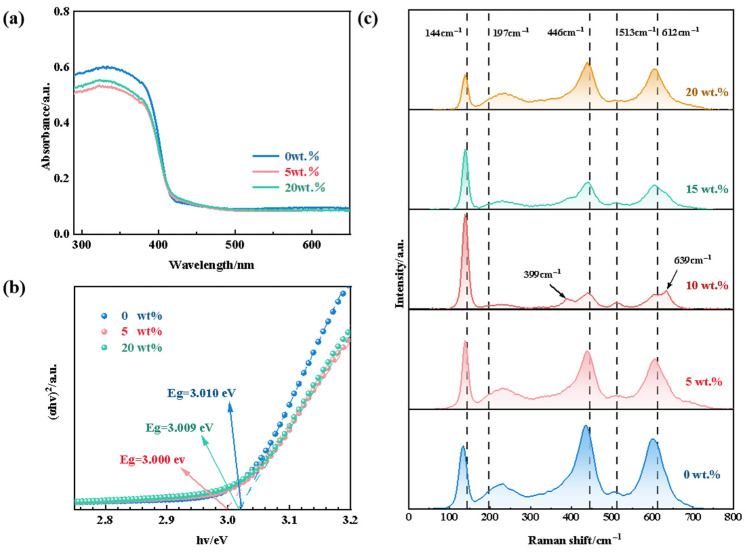
(**a**) UV–Vis DRS spectra and (**b**) energy band gap of porous ceramics with SiO_2_ contents of 0 wt.%, 5 wt.%, and 20 wt.%. (**c**) Raman spectra of porous ceramics with different SiO_2_ contents.

The absorption characteristics of the samples were examined via ultraviolet–visible (UV–Vis) diffuse reflectance spectroscopy (DRS). Figure 8a shows the DRS spectra of the prepared ceramic samples. The absorption edges for all three samples were closely aligned, with an optical response around 420 nm, which indicates effective absorption of ultraviolet light. This response is attributed to the wide band gap of titanium dioxide, which requires high-energy ultraviolet light to excite electron–hole pairs. The band gap of the ceramic samples was determined using the Tauc formula (1):

(1) $${\left(\alpha hv\right)}^{1/n}=A(hv-Eg)$$

where α is the optical absorption coefficient, h is Planck’s constant, v is the frequency (nm), A is a constant, Eg is the semiconductor bandgap (eV), and n depends on the type of semiconductor (for direct bandgap semiconductors, n = 1/2).

As shown in Figure 8b, the band gap values of the samples with SiO_2_ contents of 0 wt.%, 5 wt.%, and 20 wt.% were determined as 3.010 eV, 3.000 eV, and 3.009 eV, respectively, through tangent line extrapolation. Typical band gap values for the anatase and rutile phases are about 3.2 and 3.0 eV, respectively [62]. Therefore, for the porous ceramic sample, the band gap value was in between that of anatase and rutile, being closer to latter, in accordance with the fact that in the sample, rutile exists as the predominant phase together with anatase as the minority phase.

Figure 8c gives the Raman spectrum of the sample as a function of SiO_2_ concentration, revealing important insights into the phase transition between the anatase and rutile phases. The Raman spectra exhibited six and five active modes corresponding to the anatase and rutile phases, respectively: 144 cm^−1^, 197 cm^−1^, 399 cm^−1^, 513 cm^−1^, and 639 cm^−1^ for anatase and 144 cm^−1^, 446 cm^−1^, 612 cm^−1^, and 827 cm^−1^ for rutile. The Raman spectrum of undoped SiO_2_ showed rutile peaks at 137.10 cm^−1^, 436.62 cm^−1^, and 602.53 cm^−1^. Additionally, the spectrum featured a broad peak at 239 cm^−1^. According to Hara [63], this peak is attributed to significant lattice disorder in rutile, which could have also resulted from multi-level scattering or structural distortions. After doping with 5 wt.% SiO_2_, the intensity of the Raman peak at 140 cm^−1^ increased, whereas the intensities of the peaks at 436.62 cm^−1^ and 602.53 cm^−1^ decreased. This indicates that SiO_2_ doping causes a change in the crystal lattice, which may reduce the content of rutile. When the concentration of SiO_2_ is 10 wt.%, the highest peak appears at 138.26 cm^−1^, and the new anatase peak appears at 390.05 cm^−1^, 510.13 cm^−1^ and 634.52 cm^−1^. The appearance of these anatase peaks and the simultaneous weakening of rutile peaks at 643.62 cm^−1^ and 602.53 cm^−1^ indicate the inhibition of anatase to rutile transition. This inhibition can be attributed to the doping of SiO_2_ causing the stabilization of grain boundaries and an increase in lattice strain, thus slowing down the dynamic process of phase transition [64]. Specifically, the segregation of SiO_2_ at TiO_2_ grain boundaries not only hinders the growth of TiO_2_ grains, but also limits the formation of rutile phases, which require high grain boundary mobility. Due to the introduction of SiO_2_, grain boundary migration is inhibited, resulting in the anatase phase remaining stable over a wider temperature range, thus delaying the transition to the rutile phase [65]. Similar to the XRD pattern, further increases in SiO_2_ content promoted the A-R phase transition, where the intensity of anatase peaks reduced and eventually disappeared, whereas the rutile peaks at 138.26 cm^−1^, 230.39 cm^−1^, 439.52 cm^−1^, and 604.35 cm^−1^ became more pronounced.

**Figure 9:**
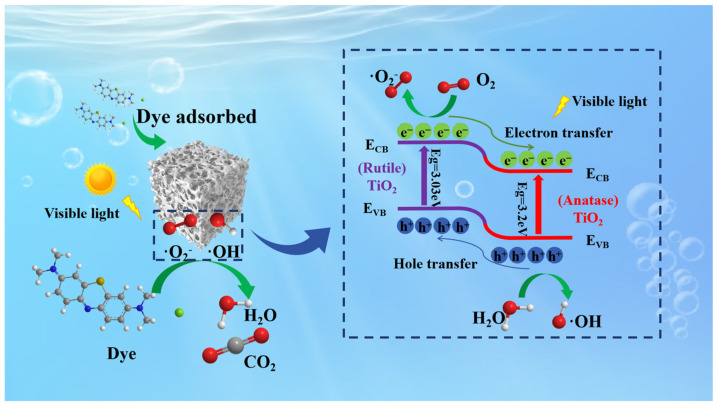
Mechanism of photocatalytic degradation of dyes using porous compound semiconductor ceramics.

According to previous studies and the analyses conducted, Figure 9 illustrates the proposed mechanism for photocatalytic degradation using Al_2_O_3_–SiO_2_–TiO_2_ porous composite semiconductor ceramics. During photocatalysis, the primary limitations to catalytic efficiency are the scarcity of active sites and the recombination of photogenerated electron–hole pairs [66]. Integrating TiO_2_ with Al_2_O_3_ and SiO_2_ insulators to create porous ceramics helps to address these challenges. Al_2_O_3_ and SiO_2_ enhance the number of catalytic sites by reducing the agglomeration of TiO_2_ particles and promoting their dispersion during calcination. They also provide a beneficial pore structure and exceptional specific surface area that support TiO_2_ photocatalysis, as demonstrated by previous scanning electron microscopy observations showing a high density of pores and a large specific surface area. Additionally, SiO_2_ inhibits the complete transformation of anatase to rutile, forming a biphasic TiO_2_ structure. Under light exposure, electrons (e^−^) in the biphasic titanium dioxide are excited and transferred to the conduction band (CB). Owing to differences in band potentials, photogenerated electrons (e_CB_^−^) migrate from the rutile CB to the anatase CB. Moreover, holes (h_VB_^+^) move from the valence band (VB) of anatase to the rutile VB to form a heterojunction between anatase and rutile. This process enhances the separation of photogenerated charges within the biphasic titanium dioxide, which significantly reduces carrier recombination. The resulting carriers (e_CB_^−^/h_VB_^+^) generate ·OH and ·O_2_^−^ radicals by reacting with O_2_ and H_2_O. Consequently, dyes adsorbed on the surface of the photocatalyst react with these ·OH and ·O_2_^−^ radicals and break down into simpler molecules to produce H_2_O and CO_2_. The potential reactions involved in the photocatalytic treatment of dye wastewater are outlined as follows (2)–(5):

(2) $${TiO}_{2}+hv\to e_{CB}^{-}+h_{VB}^{+}$$

(3) $$h_{VB}^{+}+H_{2}O\to \cdot OH+H^{+}$$

(4) $$e_{CB}^{-}+O_{2}\to {TiO}_{2}+\cdot O_{2}^{-}$$

(5) $$OH/\cdot O_{2}^{-}+Dye\to H_{2}O+{CO}_{2}$$

**Figure 10:**
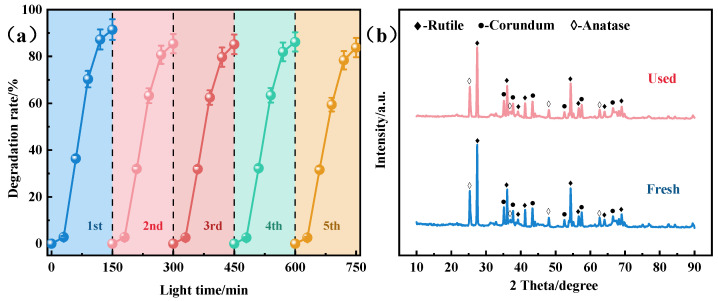
(**a**) Cyclic degradation experiments of MB dye using porous compound semiconductor ceramics. (**b**) XRD patterns before and after cycling.

To assess the potential application of the ceramic photocatalyst for methylene blue dye degradation in wastewater, the cyclic stability of the catalyst was investigated. The initial photodegradation efficiency of the catalyst was 91.5% (Figure 10a). Upon reuse, the efficiency slightly decreased to 85.49%. The third, fourth, and fifth cycles yielded degradation efficiencies of 85.2%, 86.22%, and 83.82%, respectively. The degradation rate decreased with each use, which indicated that some catalytic sites were partially blocked or inaccessible. Nonetheless, only 7.68% of the photocatalytic efficiency was lost after five cycles. Additionally, the XRD spectra (Figure 10b) revealed negligible changes in the ceramic structure after five cycles compared with the fresh ceramic. These results demonstrate that the modified porous ceramics exhibited good reusability and strong chemical stability. Therefore, the stabilization and recovery of Al_2_O_3_ and SiO_2_ in the TiO_2_ photocatalysis system are promising, and they provide more active sites for catalytic reactions while maintaining good recyclability.

## 3. Materials and Methods

### 3.1. Experimental Materials

Alumina (Al_2_O_3,_ AR) and polyvinyl alcohol (PVA, AR) were purchased from Shanghai Aladdin Biochemical Technology Co., Ltd. in Shanghai, China. Nano-TiO_2_ (anatase, AR, 15 nm), and nano-silicon oxide (SiO_2_, AR, 20 nm) was purchased from Nanjing Tianxing New Material Company. in Jiangsu Province, China. Absolute ethanol (C_2_H_5_OH, AR) was purchased from Sinopharm Chemical Reagent Co., Ltd. in Beijing, China. Methylene blue (C_16_H_18_ClN_3_S·3H_2_O, AR) was purchased from Shanghai Maclin Biochemical Technology Co., Ltd. in Shanghai, China. Corn starch (passed through a 200-mesh sieve) was purchased commercially.

### 3.2. Preparation of Porous Compound Semiconductor Ceramics

**Figure 11:**
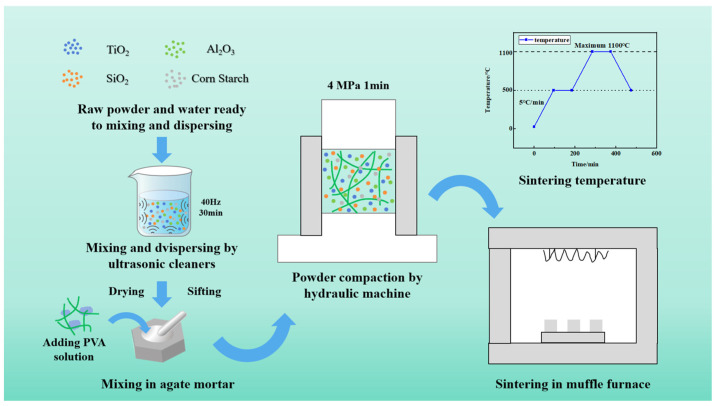
Schematic of the preparation process of porous compound semiconductor ceramics.

Figure 11 illustrates the process of preparing porous ceramics. First, raw materials were weighed according to the formula specified in Table 2. These materials were then dissolved in an equal mass of deionized water and subjected to ultrasonic dispersion for 30 min at a frequency of 40 Hz to ensure thorough dissolution. Following ultrasonic treatment, the solution was placed in a blast drying oven at 100 °C for 12 h. Subsequently, a 3 wt.% PVA solution was used as a binder. The PVA solution was mixed with the raw materials and then ground again to ensure thorough blending. The mixture was dried and sifted through a 40-mesh screen. The uniform mixture was subsequently compacted under a 4 MPa hydraulic press for 1 min to form the green body. The green bodies were placed on alumina firebricks and sintered in ambient air using a high-temperature energy-saving electric furnace. The furnace temperature was gradually increased to 1100 °C at a rate of 5 °C/min, and this temperature was maintained for 2 h. During cooling, the sample was allowed to cool naturally to room temperature. The resulting ceramic samples were then obtained.

### 3.3. Characterization

The samples were characterized using various techniques. Powder XRD was performed with a Rigaku Ultima IV diffractometer (Rigaku Corporation, Tokyo, Japan), operating at 40 kV tube voltage and 150 mA tube current, over a 10–90° 2θ range with a scan rate of 2°/min. The micromorphology of the sintered samples was analyzed using a JEOL JSM-6701F scanning electron microscope (Japan Electronics Co., Ltd, Tokyo, Japan). Porosity was measured through the Archimedes drainage method. The three-point bending strength was determined using an Instron 5567 universal testing machine, with a span of 30 mm and a loading rate of 0.5 mm/min. UV–Vis absorption spectra were recorded using a Shimadzu UV-2700 spectrophotometer (Shimadzu Corporation, Kyoto, Japan), over the 200–800 nm range, and methylene blue solution absorbance was measured at 664 nm. Raman spectroscopy was conducted using a HORIBA XploRa PLUS confocal Raman spectrometer (HORIBA, Ltd. Kyoto, Japan), with a test range of 50–2000 cm^−1^, an acquisition time of 0.5 s, and a Raman detection time of 0.5 s.

### 3.4. Visible Light Photodegradation Experimental Process

The photocatalytic properties of porous Al_2_O_3_–SiO_2_–TiO_2_ ceramics were evaluated through the photodegradation of methylene blue under visible light. A 10 mg/L methylene blue solution, adjusted to a pH of 7, was used as simulated wastewater. In a typical experiment, 100 mL of this solution was placed in a glass beaker with the ceramic sample, and the mixture was allowed to react in the dark for 30 min to achieve adsorption equilibrium. For the photocatalysis process, the sample was exposed to an 18 W LED lamp emitting at 420 nm. At 30-min intervals, 3 mL of supernatant was removed from the reaction mixture and analyzed using a UV–Vis spectrophotometer to monitor methylene blue degradation. Absorbance was measured at 664 nm. The reaction system was maintained at a temperature of 25 ± 0.5 °C with continuous ventilation.

Additionally, repeated cycle catalytic experiments were conducted to assess the recyclability of the porous Al_2_O_3_–SiO_2_–TiO_2_ ceramics. After each photocatalytic experiment, the ceramic samples were removed from the methylene blue solution, washed with deionized water and then ethanol, and subsequently dried at 70 °C for 24 h. The dried photocatalyst was then used for subsequent photocatalytic runs under the same experimental conditions. This process was repeated for five cycles.

The photocatalytic degradation rate (*η*, %) of the prepared porous ceramics was calculated using the following Equation (6):

(6) $$\eta =\frac{C_{0}-C_{t}}{C_{0}}\times 100\%$$

where *C*_0_ is the initial absorbance of the methylene blue solution, and *C*_t_ is the absorbance of the methylene blue solution at time *t* (min) during the photocatalytic process.

The photocatalytic degradation kinetics of porous Al_2_O_3_–SiO_2_–TiO_2_ ceramics were studied using a quasi-first-order kinetic model. The linear form of the pseudo-first-order kinetic model is described using Equation (7):

(7) $$-ln\frac{C_{t}}{C_{0}}=k^{\prime }\cdot t$$

where *k*′ (min^−1^) is the rate constant of the pseudo-first-order photocatalytic degradation reaction, *t* (min) is the photocatalytic reaction time in, *C*_t_ is the absorbance of the solution after timely irradiation, and *C*_0_ is the initial absorbance of the solution.

## 4. Conclusions

Porous semiconductor ceramics consisting of Al_2_O_3_-SiO_2_-TiO_2_ were successfully synthesized using the dry pressing technique, with anatase-type TiO_2_ serving as the primary catalyst and Al_2_O_3_ and SiO_2_ acting as additive materials. The findings indicated that Al_2_O_3_ could enhance the open porosity of the ceramics and inhibit the aggregation of TiO_2_, thereby increasing the catalytic site and improving the light absorption capacity. On the other hand, SiO_2_ was found to improve the bending strength of the ceramics and inhibit the conversion of anatase to rutile, which further boosted its photocatalytic activity. Consequently, at an optimal composition of 55 wt.% Al_2_O_3_, 40 wt.% TiO_2_, and 5 wt.% SiO_2_, the resulting porous ceramics displayed a methylene blue removal rate of 91.50%, and even after five cycles of testing, their catalytic efficiency remained at approximately 83.82%. These results demonstrate the exceptional photocatalytic degradation efficiency, recyclability, and reusability of the Al_2_O_3_-SiO_2_-TiO_2_ porous semiconductor ceramics, suggesting their significant potential for application in the treatment of dye wastewater.

## Figures and Tables

**Table 1:** Comparison of methylene blue degradation efficiency under visible light over different catalysts.

Photocatalysts	Catalyst Dosage	MB Initial Concentration	Light Source	Irradiation Time	Photocatalyst Efficiency	Rate Constant	Ref.
Ag-doped hydroxyapatite bio-ceramics	1 g/L	10 mg/L	Vis (624 w)	70 min	97%	6.83 × 10^−2^	[54]
SiC foam	20 g/L	1.5 × 10^−5^ mol/L	Vis (150 w)	480 min	88%	-	[55]
Ag NPs-doped TiO_2_ nanocomposite film	0.2 g/L	10 mg/L	UV (8 w)	100 min	94.6%	2.86 × 10^−2^	[56]
TiO_2_-deposited porous substrate	-	5 mg /L	UV (300 w)	240 min	50%	-	[57]
WO_3_-coated porous ceramics	-	10 mg/L	Vis (100 w)	6 h	83%	5.1 × 10^−3^	[58]
Al_2_O_3_-TiO_2_ Coatings	-	6 mg /L	Vis (4 w)	5 h	97.43%	-	[59]
P25 TiO_2_ powder	0.5 g/L	10 mg /L	UV	100 min	81.4%	-	[60]
TiO_2_-SiO_2_ powder	0.4 g/L	6 mg /L	Vis (9 w)	60 min	68%	-	[61]
Al_2_O_3_–SiO_2_–TiO_2_ porous ceramics	15 g/L	10 mg/L	Vis (18 w)	120 min	91.5%	2.09 × 10^−2^	This work

**Table 2:** Raw material ratios (wt.%).

Test Group	Titanium Oxide (wt.%)	Aluminum Oxide (wt.%)	Silicon Oxide (wt.%)	Corn Starch (wt.%)
A1	40	60	0	10
A2	45	55	0	10
A3	50	50	0	10
A4	55	45	0	10
A5	60	40	0	10
B1	40	60	0	10
B2	40	55	5	10
B3	40	50	10	10
B4	40	45	15	10
B5	40	40	20	10

## Data Availability

Data are contained within the article.
